# Addressing knowledge gaps in allergies among Syrian hospital patients: a cross-sectional study

**DOI:** 10.1038/s41598-024-53471-9

**Published:** 2024-02-05

**Authors:** Jamal Ataya, Abdullah Ismail, Jameel Soqia, Alyamama Kousa, Zein Shahoud, Rawan Alhalabi, Mazen Zaitouna

**Affiliations:** 1 Faculty of Medicine, University of Aleppo, Aleppo, Syria; 2 Faculty of Medicine, Damascus University, Damascus, Syria; 3 UMR1195, University Paris Sud, INSERM, University Paris-Saclay, Le Kremlin-Bicetre, France; 4 Service of Urology, Grand hospital of Est Francilian, Meaux, France

**Keywords:** Immunology, Public health

## Abstract

Allergies have a significant impact on health and quality of life worldwide, yet there is limited research on the awareness and knowledge of allergies. This study aimed to explore the level of awareness and knowledge of allergies among visitors in Syrian hospitals. A cross-sectional study was conducted between May and September 2022, and a standardized international questionnaire was administered to 504 visitors in three hospitals in Syria. Data analysis was performed using the Statistical Package for Social Sciences (SPSS Inc., Chicago, IL, USA) version 23. The final sample comprised 504 questionnaires with 61.7% of participants achieving an average score. Statistical analysis revealed a significant difference in knowledge of allergy scores between the group with only elementary education (M = 3.76, SD = 1.67, p = 0.011), the group with no education (M = 3.65, SD = 1.65, p = 0.006), and the group with a university education (M = 4.44, SD = 1.25). Notably, no significant differences were found between the other educational groups. A one-way ANOVA was employed to assess the effect of place of living on knowledge of allergy, but no significant differences were observed between the groups (p = 0.462). Lastly, a significant negative correlation was detected between participant age and knowledge of allergy scores (r(502) = − 0.102, p = 0.022). Allergies represent a substantial global health concern that demands attention from healthcare providers, policymakers, and the public. This study emphasizes the importance of investing in health education and awareness campaigns to enhance knowledge and comprehension of allergies, particularly individuals with lower levels of education levels. By equipping individuals with the requisite information to effectively manage their allergies, their overall health and wellbeing can be improved.

## Introduction

Allergies are hypersensitive immune reactions to harmless substances that can affect various organs and systems in the body. They are one of the most common chronic diseases in the world, affecting up to 40% of the population in some regions^1^. Allergies can have a negative impact on the quality of life, productivity, and health care costs of individuals and societies^2^.

Allergies have a significant impact on health and quality of life affecting millions of people worldwide, regardless of age and gender^1,2^. The symptoms of allergies can range from mild to severe, with some being life-threatening. Allergies are among the most prevalent chronic diseases globally, and in Syria, they are a common problem that can lead to hospitalizations and other healthcare complications. Despite the prevalence of allergies, many people do not fully understand what causes them or how to manage their symptoms effectively. These allergic reactions can be triggered by various factors, including pollen, dust, animal dander, and certain foods^3,4^. When these factors come into contact with the body, they can trigger an immune response, resulting in symptoms such as sneezing, wheezing, hives, and swelling^5,6^. Unfortunately, allergies can sometimes lead to anaphylaxis, a severe and potentially life-threatening reaction that can cause breathing difficulties and low blood pressure^2,7^. In addition to the physical symptoms of allergies, they can also have a significant impact on a person's mental health and wellbeing^8^. Allergies can be particularly challenging for children, as they may struggle to communicate their symptoms effectively or understand what is happening to their bodies^9^.

In Syria, despite the high prevalence of allergies^10^, scant attention has been paid to understanding the awareness and knowledge levels about this condition among the general public. This dearth of knowledge poses a potential risk of delayed diagnosis and treatment, with severe consequences^11^. This investigation addresses a crucial knowledge gap by assessing allergy awareness among individuals visiting primary public hospitals in Damascus, Syria. The study’s findings are set to inform and shape public health strategies that aim to enhance allergy knowledge and awareness. By doing so, it seeks to reduce the societal burden of allergies. The research underscores the urgent need for educational programs to elevate understanding and management of allergies, particularly in areas where specialized care is scarce. The insights provided by this study are instrumental in driving public health policies towards better health outcomes and mitigating the impact of allergies on society.

## Methods

### Study design

In the timeframe between May and September 2022, a descriptive cross-sectional study was conducted to assess the level of awareness and knowledge about allergy, its causes, and management among different visitors to Syrian governmental hospitals in Damascus. To collect data, the authors interviewed individuals visiting the hospitals, specifically targeting patients in waiting rooms and their accompanying companions. The data collection process utilized an electronic questionnaire created on the Google Forms platform. The interviews were carried out randomly by the researchers across various hospital departments. Participants were selected from waiting rooms and halls, and only those who provided consent were approached for participation in the study. The survey was conducted throughout a month-long period between July 15, 2022, and August 15, 2022.

### Participants and data collection

The inclusion criteria will encompass individuals aged 13–99 who are visitors to Al-Mouwasat Hospital, Al-Assad University Hospital, and Dermatology Hospital in Damascus. Participants are required to provide their consent personally or through their parents if under 18 and should be able to comprehend and respond to the questionnaire in Arabic. Exclusion criteria will include individuals with incomplete data and those who did not fully complete the questionnaire. In our study, it was crucial for participants to answer all the questions to ensure the validity and reliability of our findings. Therefore, if a participant left some questions unanswered, their data was considered ‘incomplete’ and they were excluded from the study., Exclusion criteria included individuals below the age of 13, non-Arabic speakers, and those facing significant physical or mental challenges that hindered effective participation. Refusal to provide consent and individuals who were not visitors to the specified hospitals during this period were also excluded. Participants were provided with a verbal explanation of the study’s purpose and procedures. The consent form was written in plain language and explained that participation was voluntary.

Participants were informed that they could withdraw at any time without penalty. Confidentiality was explained, and participants were assured that their names would not be associated with any data collected. Participants were also informed that they would receive no compensation for their participation. The consent form was reviewed with participants, and any questions were addressed before obtaining written consent from the participant or their parents if they are younger than 18 years old. Assistance was provided to illiterate participants, and no bias was introduced during the study. The study was conducted in accordance with the Helsinki Declaration. All experimental protocols were approved by the ethical committee in the Faculty of Medicine at Aleppo University, Syria with serial number (1468/3655).

### Questionnaire

This study utilized a questionnaire on anaphylaxis obtained the American Academy of Allergy, Asthma, and Immunology (AAAAI)^12^ and the University of Rochester Medical Center (URMC)^13^. The complete version of the questionnaire can be found in the Supplementary file. To tailor it more appropriately for patients and the general population in Syria, five questions were selected from each institution’s questionnaire. In the process of creating this questionnaire, certain questions from the original quizzes were deliberately excluded to create an effective and efficient tool. For instance, questions about allergies to certain types of shellfish or plants that are not commonly found in Syria were considered irrelevant to the Syrian population. Similarly, questions that required advanced medical knowledge or used complex terminology were omitted as they could potentially discourage respondents or lead to fatigue. The aim was to maintain a clear focus on assessing knowledge about anaphylaxis and allergies, so questions that did not directly contribute to this goal were excluded. Redundancy was also avoided by excluding questions that addressed the same concept as others. By carefully selecting the most relevant and informative questions, we aimed to maximize the educational value of the questionnaire while minimizing the time and effort required to complete it, ensuring that the questionnaire is a useful and accessible tool for the Syrian population. This questionnaire is avaible online and has been translated into Arabic. It consists of 10 questions, and responses are evaluated on a scale of three: 1 point is given for each correct answer, and 0 points for incorrect ones, resulting in a potential score range of 0–10. Additionally, an option of “I don’t know” was included to gauge the extent of public awareness regarding anaphylaxis. A higher score indicates a deeper understanding of anaphylaxis. The initial version was prepared in English and then translated into Arabic by language experts. To ensure consistency, it was then translated back into English. A pilot study involving 50 visitors was conducted, and their responses were added to the final sample. The pilot study was carried out under the same conditions and used the same methodology as the main study to ensure consistency in data collection. The data from the pilot study was included in the final sample without any modifications. This decision was made based on the premise that the pilot study data is compatible with the main study data, and its inclusion enhances the robustness of our findings. The questionnaire was reviewed based on the initial statistical study, and its reliability was assessed using Cronbach's Alpha test, which demonstrated high internal stability (0.869).

The questionnaire consists of two parts. The first section comprises eight questions about personal information, including age, educational level, gender, family status, and history of allergies. The second section includes questions about allergens, such as food (e.g., eggs, fish, milk), jewellery, and pollen. It also covers severe symptoms that allergens can cause, such as hypotension, rhinorrehea, and dyspnea. Finally, the questionnaire assesses knowledge about how to manage allergies, such as developing a treatment plan with a doctor, using adrenaline injections, and preventing exposure to allergens. The majority of the questions are closed-ended.

Each question in the questionnaire was assigned one point for a correct answer and zero points for an incorrect answer, with a total possible score of 10. The scores were categorized into three levels of knowledge: weak level from (0) to (3) were 186 (36.9%), the average level was from (4) to (6) were 295 (58.5%), and a good level from (7) to (10) were 23 (4.6%).

### Sample size

In order to calculate the necessary sample size (n), Cochran's Sample Size Formula was utilized by the research team. The formula took into consideration a 95% confidence level, represented by Z = 1.96, a margin of error of 5%, represented by e, and an estimated proportion (p) of 50% or 0.5 for the attribute of interest within the population. Additionally, q was determined as 1 − p:

$$n=\frac{{Z}^{2}pq}{{e}^{2}}$$

By using the aforementioned formula, the necessary sample size (n) for this study was determined to be 385.

### Statistical analysis

The data collected through the electronic questionnaire on Google forms was exported to Excel for analysis. Statistical analysis was carried out using SPSS Inc. version 23 software package. Chi-square and One-Way ANOVA were used to identify any correlation between the demographic variables and the level of knowledge related to allergy information’s. A p-value of less than 0.05 was considered statistically significant.

### Ethical approval and consent to participate

All experimental protocols were approved by the ethical committee in the Faculty of Medicine at Aleppo University, Syria with serial number (1468/3655). Written informed consent was collected from the participant or their parents if they are younger than 18 years old. The study complied with the principles of the Helsinki Declaration.

## Results

A total of 504 online surveys were collected. Of the participants, 47% were female and 53% were male. The mean age of the participants was 43.20 (SD = 15.89).In terms of location, 24.6% of the participants lived in Damascus,57.7% in Rif Dimashq, 3.6% in Homs, and 12.3% in East Governorates. Other sample details and characteristics are presented in Table 1**.**

**Table 1:** Semple characteristics.

Variables	Frequency	Percent
Gender		
Male	267	53.0
Female	237	47.0
Visited department		
Internal medicines departments	336	66.7
Surgical Departments	39	7.7
Clinics	37	7.3
Dermatology	92	18.3
Place of living		
Damascus	124	24.6
Aleppo	9	1.8
Homs	18	3.6
East Governorates	62	12.3
Rif Dimashq	291	57.7
Marital status		
Single	109	21.6
Married	381	75.6
Widow	8	1.6
Divorced	6	1.2
Educational level		
Elementary	187	37.1
Not educated	108	21.4
Secondary	92	18.3
College	78	15.5
Institution	39	7.7
Have you ever had an allergic disease?		
Yes, I have had		
No, I have not		
Yes, one of my relatives have		
No, also not any one in the family		

The mean test score for participants was 3.94 out of 10 (SD = 1.56). This study found that there was no statistically significant difference in knowledge of allergies between male (M = 1.89 out of 10, SD = 0.90) and female (M = 1.93, SD = 1.06) participants, t (502) = -0.69, p = 0.639).

Table 2 presents the p-values and correlations with mean scores across various variables, including gender, visited department, educational level, place of living, and age. A One-way ANOVA was performed to compare the effect of visited department or educational level on knowledge of allergy.There was a statistically significant difference between groups as determined by one-way ANOVA (F (3, 500) = 4.734, p = 0.003, and F (4, 499) = 4.24, p = 0.002, respectively). A Tukey post hoc test revealed that knowledge of allergy was statistically significantly lower in the group who visited internal medicine departments (M = 3.79, SD = 1.53, p = 0.003) compared to the group who visited dermatology department (M = 4.41, SD = 1.65). There was no statistically significant difference between other groups. Additionally, knowledge of allergy was statistically significantly lower in the group who had only elementary education (M = 3.76, SD = 1.67, p = 0.011) and in the group who did not have education (M = 3.65, SD = 1.65, p = 0.006) compared to the group who had a university education (M = 4.44, SD = 1.25). There was no statistically significant difference between other groups. A one-way ANOVA was performed to compare the effect of place of living on knowledge of allergy, and there was no statistically significant difference between different groups (p = 0.462). Finally, there was a significant negative correlation between the age of the participants and knowledge of allergy (r (502) = − 0.102, p = 0.022).

**Table 2:** Shows p-values and correlations with mean scores across variables.

Variable	Comparison	Mean (M)	Standard deviation (SD)	T/F value	P-value
Gender	Male vs female knowledge of allergies	Males: 1.89, females: 1.93	Males: 0.90, females: 1.06	t (502) = − 0.69	0.639
Visited department	Internal medicine vs dermatology	Internal medicine: 3.79, dermatology: 4.41	Internal medicine: 1.53, dermatology: 1.65	F (3,500) = 4.734	0.003
Educational level	Elementary/none vs university	Elementary: 3.76, none: 3.65, university: 4.44	Elementary: 1.67, none: 1.65, university: 1.25	F (4,499) = 4.24	0.002
Place of living	Different groups	–	–	–	0.462
Age	Correlation with knowledge of allergy	–	–	r (502) = − 0.102	0.022

## Discussion

The majority of participants in this investigation showed an average score when evaluating their understanding of allergies. Compared to the lower scoring groups, only a few groups exceeded the average. These results demonstrate an apparent deficiency in the delivery of healthcare education about allergies. Our study, which was the first of its kind in Syria, was implemented, which included Al-Assad University Hospitals, Al-Mouwasat, and the Dermatology Hospital in Damascus.

The aim of the study was to evaluate visitors' knowledge of allergy, its causes, and management. At first, only a few patients were capable of providing the required response regarding the time necessary for symptoms to emerge, as a few of them possess knowledge concerning the severity of anaphylaxis symptoms. This percentage was significantly lower than the outcomes of a study conducted in Brazil, where more than half of participants, were able to provide correct answers^14^. The disparity in results may be attributed to insufficient attention towards allergy sufferers in society, particularly in poor rural and eastern cities, which constitute the majority of the research participants. This issue could be resolved through health education via various media and health centres to prevent potentially fatal consequences in the event of an allergic shock, especially since only a few participants were aware of the most serious symptoms of anaphylactic shock. A study evaluating dentists' knowledge of anaphylactic shock revealed that none of the participating dentists were aware of the signs and symptoms of anaphylactic shock, as their academic and scientific awareness was inadequate^15^. In terms of awareness of conjunctivitis as an allergy symptom, the majority of participants recognized it as such, while a low percentage did not, and a few of them were uncertain, indicating a relatively high level of awareness regarding ophthalmic allergy symptoms. This may be due to the obvious signs of allergies, such as redness and watering of the eyes, and the influence of social media on the dissemination of allergy knowledge. These findings are consistent with a study conducted in Ghana, which assessed the level of knowledge and awareness of ocular allergy among undergraduate students of public universities^16^. The study revealed that less than half of the students were aware of ocular allergy, but the overall level of knowledge was generally low^16^.

Upon completion of the symptom inquiry, we proceeded to investigate the prevailing triggers of allergic reactions, particularly in light of documented cases of anaphylaxis resulting from inhalation of allergens emanating from various sources such as fish, shellfish, seeds, soybeans, cereal grains, eggs, milk, and other edibles via airborne exposure, such as flour and vapours during cooking or roasting^17^. Interestingly, when surveying participants regarding the likelihood of anaphylactic episodes from ordinary foods like milk, eggs, and fish, the most of them provided the correct response, owing to the prevalence of food allergies attributed to these traditional staples. Analogously, primary care physicians in Turkey were questioned about the three most common food allergies, with most identifying eggs, milk, and strawberries as the culprits^18^. Regarding other allergens, we acknowledge the growing public concern surrounding fine dust, which has become a pressing societal issue^19^. Encouragingly, more than half of our participants correctly identified dust and nickel metals as potential allergens, possibly attributed to the arid environmental conditions prevalent in the eastern provinces and countryside where the majority of the respondents reside. However, only a few number recognized the bed as a likely habitat for dust mites, presumably due to the lack of public attention devoted to these minuscule arthropods. In contrast, a study evaluating the efficacy of dust mite control demonstrated that families of children who underwent skin examination by an allergist exhibited greater awareness and more adept control of environmental factors that contribute to dust mite proliferation^20^.

The findings of this study suggest that regular monitoring of medical centres is crucial for detecting symptoms and increasing awareness of unfamiliar causes, such as dust mites, as well as providing appropriate management strategies for allergic cases. Additionally, our investigation found that more than half of individuals correctly answered a question about treating anaphylaxis with medication, which suggests a lack of medical awareness among the general population. Similarly, only a few of respondents knew how to manage their condition if at risk of anaphylaxis. The level of knowledge of nursing students was significantly higher than that of the general population on the same questions^21^.

The study found that the average test score for participants was 3.94 out of 10 and this shows a disturbing lack of knowledge about allergies among the general population. This is corroborated by a cross-sectional study among restaurant staff in Germany, which found that food allergy knowledge was suboptimal, with only a small percentage of respondents correctly naming three common food allergens^22^. Similarly, a study that examined food allergy knowledge and attitudes among kindergarten teachers found an average score of more than half on the knowledge assessment^23^. These findings suggest that the low level of allergy knowledge is not isolated to our study’s population but is a widespread issue. Moreover, the absence of a statistically significant difference between the acceptance rates of male and female participants for permanent review of medical centers indicates that anaphylaxis is a universal concern. This aligns with an international survey on knowledge and attitudes towards allergic rhinitis, which found differing perceptions of allergic rhinitis among patients and physicians, suggesting a need for improved education and communication^24^. The survey’s results emphasize that both healthcare providers and the public require better allergy education to manage and prevent allergic diseases effectively. Our study highlights the critical need for comprehensive allergy education programs that cater to the general public based on these comparisons. Such programs should aim to improve the understanding of allergies and anaphylaxis, equip individuals with the knowledge to recognize and respond to allergic reactions, and ultimately contribute to better health outcomes for those affected by allergies.

Expanding on the initial statement, it is important to consider that while personal experience with a condition like allergies might intuitively lead to better knowledge, research indicates that this is not a universal truth. In a study that assessed food allergy knowledge among restaurant staff it was discovered that personal experience with food allergies did not correlate with a higher level of knowledge^23^. This could be due to the complex nature of allergies, which encompasses a wide range of symptoms and triggers, making it challenging for individuals to fully understand the condition without comprehensive education. Moreover, a study exploring knowledge-user experiences in integrated knowledge translation for food allergies emphasized the importance of meaningful interactions between scientists and knowledge-users, such as patients and policy advocates^25^. Collaborative efforts are necessary to enhance the understanding of allergies among those affected by the condition as suggested by this study. Additionally, a cross-sectional study in Kuwait assessed the knowledge, attitudes, and beliefs of kindergarten teachers regarding food allergies. According to the results, the participants scored an average of 52.2% on the food allergy knowledge assessment, which indicates a moderate level of understanding^26^. Teachers with prior training in food allergies scored higher than those without, highlighting the impact of education on knowledge levels. These studies collectively suggest that while personal experience with allergies can provide some insights into the condition, it is not sufficient to ensure a comprehensive understanding. Educational interventions, such as training programs and collaborative research initiatives, are crucial in bridging the knowledge gap and ensuring that both patients and the general public are well-informed about allergies and their management.

Finally, our study revealed a negative correlation between age and knowledge of allergy, which may be explained by younger individuals' greater access to diverse sources of information and the widespread dissemination of medical awareness through social media. Therefore, promoting medical awareness through various channels such as schools, social media, and advertisements could be an effective strategy for improving the general population's knowledge of anaphylaxis and related conditions.

The difference in allergy knowledge observed between different educational levels observed in this study is consistent with findings from other research. For instance, a cross-sectional study conducted among restaurant staff in Germany found that food allergy knowledge was suboptimal, and certain determinants, such as the level of school education, influenced knowledge levels^23^. This aligns with our study’s observation that individuals with university education had a higher awareness of allergies. The German study’s insight on the determinants of knowledge can be used to guide targeted educational interventions, emphasizing the importance of comprehensive allergy education across all levels of society. Moreover, the lack of significant differences in allergy knowledge based on geographic location suggests that educational efforts should be uniformly distributed across various regions. This is in contrast to a study comparing children with and without food allergy experience, which did not find a significant difference in knowledge levels between the two groups^22^. The need for structured educational programs is supported by the fact that personal experience with food allergies does not necessarily translate into better knowledge, further supporting the need for structured educational programs.

In terms of department-specific knowledge, our study highlights the importance of specialized educational programs within hospital departments. Patients visiting dermatology departments showed significantly higher allergy knowledge, likely due to the direct association between skin symptoms and allergic reactions. This suggests that other departments, such as internal medicine, could benefit from incorporating more allergy-related discussions into patient interactions. Providing healthcare professionals with department-specific training and enhancing allergy communication ensures that patients receive comprehensive information regardless of the department they visit. The emphasis on department-specific knowledge in our study is supported by the findings of the World Allergy Organization, which conducted a multicentre study to assess allergy knowledge and perceptions among the population. According to their research, the level of education affects allergy awareness and participants are more likely to receive treatment from an allergologist when they experience allergy symptoms^21^. This suggests that specialized training in allergy care within hospital departments can significantly impact patient outcomes. Similarly, a systematic review on infection prevention and control among healthcare workers highlighted the importance of knowledge and compliance with guidelines, which has critical implications for patient protection and the care environment^27^. While this study focused on infection control, the principles of department-specific knowledge and training are applicable to allergy education as well. Furthermore, a descriptive report on establishing a protocol and policy for the safety of patients with food allergies in a hospital setting emphasized targeting specific hospital processes and sectors involved in patient care^28^. By incorporating more allergy-related discussions into patient interactions, non-dermatological departments, such as internal medicine, can enhance their allergy knowledge, as suggested by our study.

## Conclusion

In conclusion, this study emphasizes the need for increased health education and awareness campaigns to improve the limited knowledge and awareness of allergies among patients in Syrian hospitals. Efforts should be targeted towards individuals with lower levels of education, who demonstrated significantly lower levels of allergy knowledge. Improving public health outcomes and quality of life can be achieved by providing individuals with the necessary information to manage their allergies effectively.

### Supplementary Information

Supplementary Information.

## Data Availability

All data generated or analysed during this study are included in this published article.
