# Supplementary material for: Addressing knowledge gaps in allergies among Syrian hospital patients: a cross-sectional study
Source: Sci Rep. 2024 Feb 5;14:2938. doi: 10.1038/s41598-024-53471-9 (PMC10844623; doi:10.1038/s41598-024-53471-9)
Supplement: Supplementary file 1 — Supplementary Information. [file 41598_2024_53471_MOESM1_ESM.docx]

| **Questionnaire:** The questions evaluate the level of knowledge about allergies. | | | |
| --- | --- | --- | --- |
|  | | **Count** | **Column N %** |
| **1.Symptoms of anaphylaxis can occur:** | Short period of touching | 279 | 55.4% |
|  | Long period of touching | 88 | 17.5% |
|  | I don’t know | 73 | 14.5% |
|  | **Both are correct *** | 64 | 12.7% |
| **2.An anaphylactic reaction can be as simple as developing a rash after exposure to an allergen.** | Right | 386 | 76.6% |
|  | **Wrong *** | 83 | 16.5% |
|  | I don’t know | 35 | 6.9% |
| **3.Anaphylaxis can occur from eating common foods such as milk, eggs, or shellfish.** | **Right *** | 390 | 77.4% |
|  | Wrong | 81 | 16.1% |
|  | I don’t know | 33 | 6.5% |
| **4.Anaphylaxis always requires medical treatment.** | Right | 300 | 59.5% |
|  | **Wrong *** | 176 | 34.9% |
|  | I don’t know | 28 | 5.6% |
| **5.The most severe form of allergic reaction is called anaphylaxis. Which symptoms might happen with this?** | Difficulty in Breathing | 183 | 36.3% |
|  | Blood pressure decreasing (Hypotension) | 76 | 15.1% |
|  | Rhinorrhea | 22 | 4.4% |
|  | **All of the above *** | 74 | 14.7% |
|  | I don’t know | 149 | 29.6% |
| **6.If you are at risk for anaphylaxis, the best way to manage your condition is:** | Avoiding allergic materials | 293 | 58.1% |
|  | Make a plan to manage allergic cases | 40 | 7.9% |
|  | Always carry an epinephrine shot | 17 | 3.4% |
|  | **All of the above *** | 107 | 21.2% |
|  | I don’t know | 47 | 9.3% |
| **7.Which of these body systems causes allergic reactions?** | **The immune system *** | 245 | 48.6% |
|  | Endocrine | 30 | 6% |
|  | Aautonomic nervous system | 18 | 3.6% |
|  | I don’t know | 211 | 41.9% |
| **8.An allergen is anything that triggers an allergic response. Which of these could be an allergen?** | Dust | 101 | 20.0% |
|  | Food | 36 | 7.1% |
|  | Nickel | 2 | 0.4% |
|  | **All of the above *** | 321 | 63.7% |
|  | I don’t know | 44 | 8.7% |
| **9.Dust mites are a common trigger for indoor respiratory allergies. Where are you most likely to find them in the home?** | Curtains | 14 | 2.8% |
|  | Carpet | 146 | 29% |
|  | **Bed *** | 17 | 3.4 % |
|  | All of the above | 286 | 56.7% |
|  | I don’t know | 41 | 8.1% |
| **10.Allergies can cause conjunctivitis:** | **Right *** | 385 | 76.4% |
|  | Wrong | 52 | 10.3% |
|  | I don’t know | 67 | 13.3% |
